# Severity of Occlusal Disharmonies in Down Syndrome

**DOI:** 10.1155/2012/872367

**Published:** 2012-08-15

**Authors:** Danielle Bauer, Carla A. Evans, Ellen A. BeGole, Larry Salzmann

**Affiliations:** ^1^Private Practice, Wheaton, IL, USA; ^2^Department of Orthodontics, University of Illinois at Chicago, 801 South Paulina Street, MC 841, Chicago, IL 60612-7211, USA; ^3^Department of Pediatric Dentistry, UIC College of Dentistry, 801 South Paulina Street, MC 850, Chicago, IL 60612, USA

## Abstract

*Objective*. To quantify the severity of malocclusion and dental esthetic problems in untreated Down syndrome (DS) and untreated non-Down syndrome children age 8–14 years old using the PAR and ICON Indices. *Materials and Methods*. This retrospective study evaluated pretreatment study models, intraoral photographs, and panoramic radiographs of 30 Down syndrome and two groups of 30 non-Down syndrome patients (private practice and university clinic) age 8–14 years. The models were scored via PAR and ICON Indices, and descriptive characteristics such as Angle classification, missing or impacted teeth, crossbites, open bites, and other dental anomalies were recorded. *Results*. The DS group had significantly greater PAR and ICON scores, as well as 10 times more missing teeth than the non-DS group. The DS group possessed predominantly Class III malocclusions, with the presence of both anterior and posterior crossbites in a majority of the patients. The non-DS group had mostly Class I or II malocclusion with markedly fewer missing teeth and crossbites. The DS group also had more severe malocclusions based upon occlusal traits such as open bite and type of malocclusion. *Conclusion*. The DS group had very severe malocclusions, while the control group from the university clinic had more severe malocclusions than a control group from a private practice.

## 1. Introduction

Down syndrome (DS) was first described in 1866 by John Langdon Down and affects 1 in every 600–1000 live births; DS is the most common genetic cause of intellectual disabilities [1]. Trisomy of chromosome 21 is the most common cause for DS, accounting for approximately 95% of all DS cases [1]. The life span of DS individuals is increasing, and so is the need for dental and orthodontic care.

Craniofacial anomalies accompany systemic manifestations along with varying degrees of lack of normal intellectual development. Specific features of Down syndrome include reduced muscle tone, hypoplastic maxilla, compromised immune system, mouth breathing, mental impairment and malocclusion. These individuals display characteristic facial features, including oblique eye fissures, protruding tongue, Brushfields spots, a flat nasal bridge, and hypotonia [2]. In an anthropometric study by Allanson et al. [3], the measurements of head length from temporale to temporale were significantly smaller, along with ear length and nasal protrusion. Striking orofacial features in a DS patient are an underdeveloped midface, resulting in a flattened bridge of the nose and bones of the midface, and the appearance of a prognathic mandible, together causing Class III dental and skeletal relationships.

There have been many studies on the relationship between the cranial base and facial skeleton [4]. Hopkin et al. [5] reported that the articulare-sella-nasion angle (Ar-SN) was smaller in skeletal Class III than Class II patients, and a decrease in flexion of the cranial base was considered to be one of the etiologic factors of a skeletal Class III pattern. It has also been demonstrated that the flexure of the cranial base (nasion-sella-basion) plays a role in rotating the maxilla, creating excess posterior maxilla growth and anterior rotation of the anterior maxilla, thus creating an open bite. Fischer-Brandies [6] analyzed craniofacial development in DS patients ages 0–14 years and compared them to a control group consisting of age-matched healthy children. In the study, it was noted that the midface area and the anterior cranial base (sella-nasion) were underdeveloped in the youngest age group (0 to 3 months). The length deficit increased up to the 14th year of life. The cranial base flexure angle (nasion-sella-basion) was obtuse, indicating a flat cranial base, which correlates with the Hopkins's earlier study of interactions between cranial base and maxillo-mandibular relationships.

The dental anomalies seen often are anterior open bite, narrow maxilla, a seemingly prognathic mandible, oligodontia, periodontal disease, tooth agenesis, taurodontism, microdontia, altered eruption of primary and permanent dentition, and malalignment [7]. In a study by Ondarza et al. [8], the sequence of eruption of deciduous teeth was compared between a DS sample and a control sample. It was found that emergence of the maxillary central and lateral incisors, the maxillary left first molar, and the mandibular lateral incisors was significantly delayed, by two to three years in the DS patients. In some cases, the full deciduous dentition was not present until 5 years of age. There is also a high rate of congenitally missing teeth in both the primary and permanent dentitions [9, 10]. Russell and Kjaer [11] found that individuals with DS have an occurrence of agenesis that is roughly 10 times greater than in the general population with a higher frequency in males than in females, more common in the mandible than the maxilla, and more often on the left side than the right. The most significant difference noted was the relatively common congenital absence of mandibular incisors. It has also been noted that bruxism is quite prevalent among DS patients. López-Pérez et al. [12] confirmed this observation and found that 42% of the DS patients were bruxing. The bruxism is thought to have a multifactorial etiology and differs among regions, for example, the authors report it to be higher among the US population.

**Figure 1:**
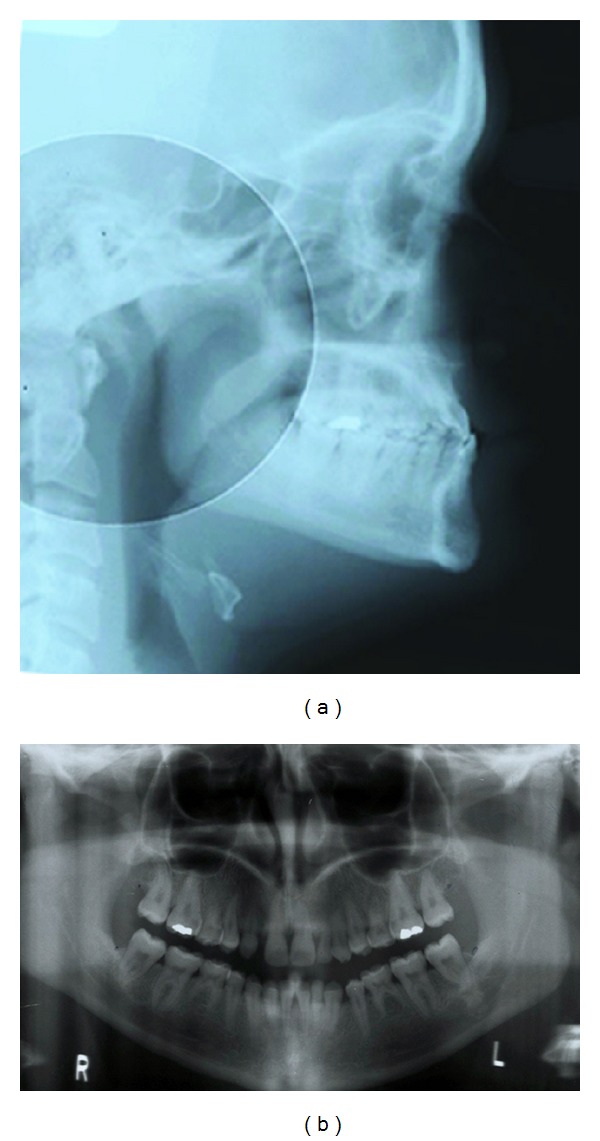
(a,b) Lateral cephalometric and panoramic radiographs of an adolescent with Down syndrome show typical skeletal disharmony, malocclusion, and permanent tooth agenesis.

Given these characteristics, it is evident that DS patients are in need of orthodontic care to treat their malocclusions. Since many DS individuals are functioning normally in society, orthodontic treatment may also improve self-esteem [2]. Medical practitioners have employed advanced medical treatment modalities [13], to benefit DS patients; however, oral health care providers, such as orthodontists, have been slow to include DS patients in their practices and to relate the orofacial anomalies to other medical conditions [14]. While many orthodontists are aware of the dentofacial complexities of DS patients, they may not recognize the degree of complexity and the need for treatment of these patients (Figure 1).

## 2. Materials and Methods

This was a retrospective study analyzing pretreatment orthodontic records of DS and non-DS patients in the age group of 8–14 years. Three groups of 30 subjects were selected randomly.

1. Group 1 (DS): children aged 8–14 years old who have DS with no other syndromes or cleft lip and palate. The records were from a private office.
2. Control group 1: subjects group were chosen from a pool of orthodontic patients in the same private office, age 8–14 years old with no significant medical history, no genetic malformations, no cleft lip or palate, and no previous surgery involving the head and neck.
3. Control group 2: subjects were chosen from a pool of orthodontic patients in a university orthodontic clinic age 8–14 years old with no significant medical history, no genetic malformations, no cleft lip or palate, and no operations involving the head and neck.

Each set of records was scored by one person who had successfully completed a PAR calibration course. The judge recorded the peer assessment rating (PAR) and index of complexity, outcome, and need for treatment (ICON) scoring, as well as the Angle classification (Class I, II or III), presence of a crossbite (posterior or anterior), missing teeth, impacted teeth, and anomalies in shape or size of teeth. Panoramic radiographs were used to confirm missing and impacted teeth. Also, abnormally shaped roots were recorded. The PAR index has been used as a tool to provide a single summary score for all the occlusal anomalies which may be found in a malocclusion. The total score represents the degree to which a person's occlusion deviates from normal alignment. The PAR Index is comprised of the scores of 5 individual traits: anterior alignment of the dentition, right and left buccal segment relationship, overjet, overbite, and midline discrepancy. A high PAR score indicates deviation from normal occlusion [15]. Another index of malocclusion was adapted in 2000 to assess treatment need, complexity, and improvement. The ICON takes into consideration a dental esthetic component, with the rationale that patients usually seek orthodontic treatment for esthetic improvements [16].

## 3. Results

After each model was measured using PAR and ICON scoring, the components were totaled and multiplied by the appropriate weightings to yield the total PAR and ICON scores. The mean PAR and ICON scores with their standard deviations are seen in Table 1. One-way ANOVA was constructed to observe overall differences, followed by Scheffé tests to evaluate pairwise comparisons of the groups. These can be seen in Tables 2 and 3. Control 1 represents the control group from the private practice, and Control 2 represents the sample taken from the university clinic.

**Figure 2:**
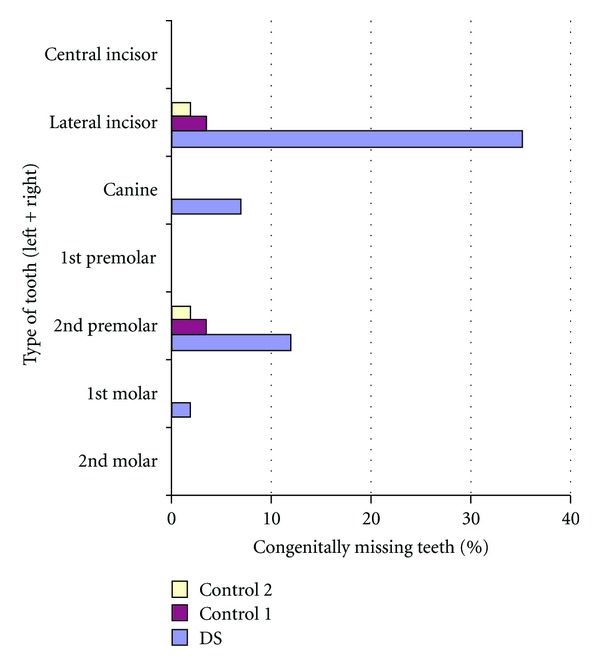
Comparison of missing maxillary teeth between the three groups.

**Figure 3:**
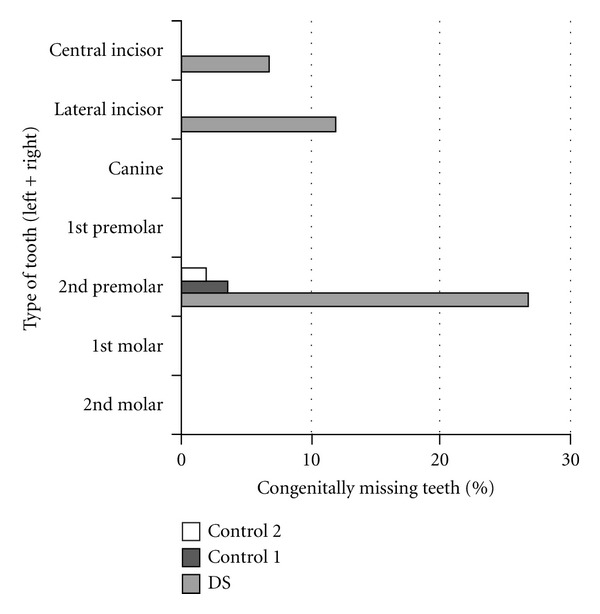
Comparison of missing mandibular teeth between the three groups.

**Figure 4:**
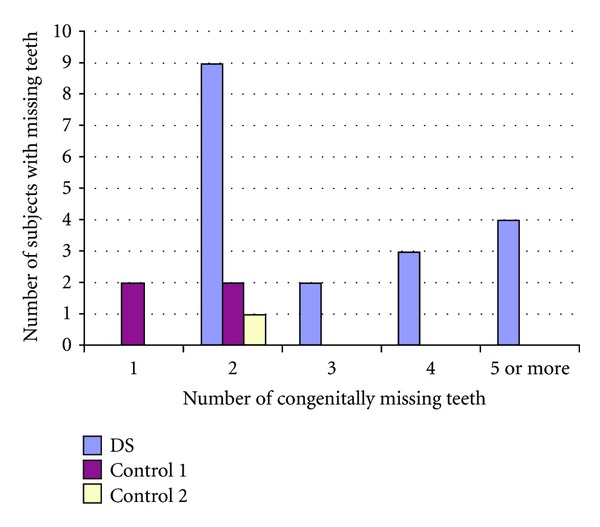
Distribution of subjects with multiple missing teeth.

**Figure 5:**
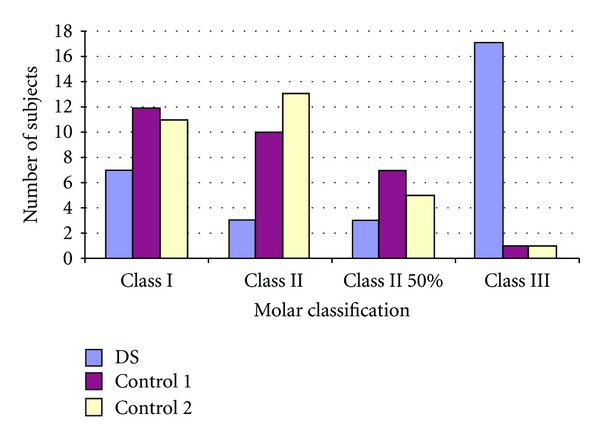
Distribution of molar classification.

Tables 4 and 5 list the number of missing and impacted teeth in each group. Figures 2 and 3 show the comparisons of missing teeth in the maxilla and mandible in each group.  Figure 4 shows the number of subjects with multiple missing teeth. Each study model was evaluated for the molar classification based on Angle's Classification. The distribution of molar classification in each group is seen in Figure 5. Anomalies such as peg-shaped teeth, abnormally shaped roots, anterior crossbite, posterior crossbite, openbite, and bruxism were recorded. These traits can be seen in Table 6.

## 4. Discussion

The mean PAR score, as seen in Table 1, for the DS group was 35.97; for Control 1 it was 17.73, and for Control 2 it was 26.60. For the PAR scores, significant differences were found between the DS and Control 1, DS and Control 2, and Control 1 and Control 2 groups, per the Scheffé test in Table 2. In the buccal occlusion section, there were fairly high scores for DS and Control 2 groups due to the presence of posterior crossbites. According to Hopkin et al. [5], the palatal vault differs in individuals with DS than the normal population in that it is narrow and V-shaped arch. The narrow maxilla and a normal transverse dimension in the mandible is a possible etiology of a posterior crossbite, either unilaterally or bilaterally. This also agrees with a study by Bhagyalakshmi et al. [17] who found that mean height of the palatal vault in DS is significantly higher than in the normal population. In a study by Uong et al. [18], magnetic resonance imaging was used to measure soft and hard tissues that contribute to airway. They found that soft tissue measurements such as the tongue and soft palate in DS were comparable in size to normal children of the same age, but the hard palate was reduced in width and depth. Therefore, the general underdevelopment of the maxillary and palatine bones seems to crowd out the tongue, requiring it to protrude and not allowing it to develop the maxilla as it does with normal tongue posture.

The majority of the DS group had a Class III malocclusion (Figure 4). In Control 1 and Control 2, most were either near Class I or Class II. That many DS patients possess Class III malocclusions that agrees with other studies, including Fink et al. [19], Oliveira et al. [20], Desai [7], and De Moraes et al. [21]. Twenty-one of the 30 DS patients had negative overjet (Table 6). The negative overjet, with or without open bite, can be related to the posture of the tongue, since it tends to protrude, thus pushing the lower incisors forward [7].

The ICON scores included components similar to the PAR index. As seen in Table 1, the mean ICON score for the DS group was 60.37, for Control 1 it was 43.27, and for Control 2 it was 46.93. There were statistically significant differences between the DS and each control group, but no significant differences between Control 1 and Control 2, per the Scheffé test in Table 3. This is probably due to higher scores in the esthetic component in the DS group due to larger number of crossbites and open bites. According to Richmond et al. [15], the cutoff for treatment of an ICON score is 43 weighted points. In the DS group, 23 of 30 patients had greater than 43, with the highest score 111 and many others in the range of 60 to 80. Only 7 DS patients had a weighted score less than 43. In Control 1, 13 of 30 patients had a weighted score of more than 43, with the highest being 67 and the lowest 13. In Control 2, 17 of 30 had a weighted score of greater than 43, with the highest being 79 and the lowest 26.

As seen in Tables 4 and 5, the DS group had a significantly greater number of missing and impacted teeth than both of the control groups. The teeth were confirmed to be congenitally missing by evaluating subsequent panoramic radiographs and checking with the office manager to ensure that the teeth were not extracted. Third molars were excluded from the research because many of the patients studied were only 8 years of age and the presence of the third molar tooth buds may not appear on a panoramic radiograph at that age.

Anomalies other than missing or impacted teeth were noted in each group and can be seen in Table 6. Thirty-three percent of DS patients displayed bruxism as judged by clinical wear of the teeth on the photos and study models. In the control groups, Control 1 showed 10% and Control 2 showed 6.7%. An anterior open bite was present in 5 of the 30 DS patients (16.7%), which was also a finding of Brown and Cunningham [22]. Both control groups contained 1 patient (3.3%) with an open bite. The range of an open bite in the normal population is 5 to 7% [23]. Oliveira et al. [20] diagnosed 21% of DS patients with an anterior open bite. Quintanilla et al. [24] found the average open bite to be −1.1 mm, ranging from −5.5 to 2 mm. Twenty-three percent of the DS patients had either peg laterals-or conical-shaped teeth or roots. Tooth size in the permanent dentition in the DS population has been relatively well-documented, and demonstrates a reduction in tooth size, mainly in the mesiodistal width [22, 25]. The reduced tooth size, along with tongue posture and missing teeth, contributes to interdental spacing in the DS population. Oredugba [26] found that 14% of DS patients had peg maxillary lateral incisors. Tooth transposition, mainly involving canines and premolars, is a relatively uncommon finding in the normal population, typically about 0.1 to 0.3% [27].

Papadopoulos et al. [28] performed a meta-analysis of the literature and found the prevalence of tooth transposition to be 0.33%, with occurrence more common in the maxilla than in the mandible, possibly due to the density of bone in the mandible not allowing the tooth buds to migrate. In this study, two of the 30 (6.7%) of the DS patients had a transposition, both of the canine and first premolar with one on the left and one on the right. In both cases, the transposition was related to an anomaly, with one having peg lateral incisors, and the other having a missing lateral incisor on the same side as the transposition. The prevalence of anterior crossbite in the DS group was 67%. This is higher than the prevalence reported in Oliveira et al. [20], where the authors found 33% had anterior crossbites. This could be due to the fact that in this study, a crossbite of more than one tooth was recorded and in the Oliveira et al. [20] study, they may have recorded it only if all anterior teeth were in crossbite. Quintanilla et al. [24] observed an anterior crossbite in 38.4% of DS patients with lower incisor protrusion in 84.6%. As discussed previously, evidence of a posterior crossbite was noted in 76% of DS patients. Of this 76%, 46% had a unilateral crossbite and 30% had a bilateral posterior crossbite. Jensen et al. [23] found bilateral crossbites in 68% of their DS group, which closely resembles our study. Brown and Cunningham [22] found a slightly lower prevalence of 56%, either unilateral or bilateral.

## 5. Conclusions

- The DS group had a very severe malocclusions as judged by the PAR and ICON scoring, as well as the descriptive aspects of the occlusion, such as open bite, malocclusion type, and missing teeth.
- The prevalence of missing teeth in the DS group was approximately 10 times more common than in the control group.
- Anterior and posterior crossbites were more prevalent in DS group.
- Overall, the DS group had the highest PAR and ICON scores, while the group from the university clinic had more severe malocclusions than a control group from a private practice.

## Figures and Tables

**Table 1:** PAR and ICON scores.

Group	Mean PAR scores ± S.D.	Mean ICON scores ± S.D.
DS	35.97 ± 9.68	60.37 ± 19.61
Control 1	17.73 ± 9.41	43.27 ± 14.07
Control 2	26.60 ± 12.25	46.93 ± 13.79

**Table 2:** PAR one-way ANOVA with pairwise Scheffé comparisons between groups.

Group	Mean difference	*P*-value	CI
DS-control 1	18.23	0.000	11.47–25.00
DS-control 2	9.37	0.004	2.60–16.13
Control 1-control 2	8.87	0.007	2.10–15.63

(*F* = 22.5, *P* ≤ 0.00).

**Table 3:** ICON one way ANOVA with pairwise Scheffé comparisons between groups.

Group	Mean difference	*P*-value	CI
DS-Control 1	17.10	0.000	6.78–27.42
DS-Control 2	13.43	0.007	3.11–23.75
Control 1-Control 2	3.67	0.677	6.65–13.99

(*F* = 9.4, *P* ≤ 0.00).

**Table 4:** Number and percentage of missing teeth by group.

No. tooth	DS	Control 1	Control 2
UR6	1 (3.3%)	0	0
UR5	4 (13.3%)	1 (3.3%)	1 (3.3%)
UR3	2 (6.7%)	0	0
UR2	10 (33.3%)	1 (3.3%)	0
UL2	11 (36.7%)	1 (3.3%)	1 (3.3%)
UL3	2 (6.7%)	0	0
UL5	3 (10%)	1 (3.3%)	0
LL5	7 (23.3%)	1 (3.3%)	1 (3.3%)
LL2	3 (10%)	0	0
LL1	2 (6.7%)	0	0
LR1	2 (6.7%)	0	0
LR2	4 (13.3%)	0	0
LR5	9 (30%)	1 (3.3%)	0

**Table 5:** Number and percentage of impacted teeth by group.

No. Tooth	DS	Control 1	Control 2
UR5	1 (3.3%)	1 (3.3%)	0
UR4	2 (6.7%)	0	0
UR3	4 (13.3%)	3 (10%)	1 (3.3%)
UR2	1 (3.3%)	0	0
UR1	0	1 (3.3%)	0
UL1	0	0	1 (3.3%)
UL2	2 (6.7%)	0	0
UL3	4 (13.3%)	4 (13.3%)	3 (10%)
UL4	1 (3.3%)	0	0
UL5	1 (3.3%)	1 (3.3%)	0
LL3	0	0	1 (3.3%)
LR3	1 (3.3%)	0	0

**Table 6:** Number and percentage of clinical dental characteristics.

Clinical characteristics	DS	Control 1	Control 2
Bruxism	10 (33.3%)	3 (10%)	2 (6.7%)
Open bite	5 (16.7%)	1 (3.3%)	1 (3.3%)
Peg teeth/shape anomalies	7 (23.3%)	4 (13.3%)	3 (10%)
Transposition	2 (6.7%)	0	0
Anterior crossbite	20 (66.7%)	5 (16.7%)	15 (50%)
Posterior crossbite			
>1 tooth	23 (76.7%)	5 (16.7%)	11 (36.7%)
1 tooth	2 (6.7%)	3 (10%)	5 (16.7%)
